# Current Molecular-Targeted Therapies in NSCLC and Their Mechanism of Resistance

**DOI:** 10.3390/cancers10070224

**Published:** 2018-07-04

**Authors:** Zachary Schrank, Gagan Chhabra, Leo Lin, Tsatsral Iderzorig, Chike Osude, Nabiha Khan, Adijan Kuckovic, Sanjana Singh, Rachel J. Miller, Neelu Puri

**Affiliations:** Department of Biomedical Sciences, University of Illinois College of Medicine at Rockford, Rockford, IL 61107, USA; zacharyschrank15@augustana.edu (Z.S.); gaganbhu@gmail.com (G.C.); llin34@uic.edu (L.L.); tsatsral87@gmail.com (T.I.); cosude2@uic.edu (C.O.); nkhan28@uic.edu (N.K.); bosnian16@gmail.com (A.K.); ssingh92@uic.edu (S.S.); rjmille2@uic.edu (R.J.M.)

**Keywords:** lung cancer, molecularly-targeted therapies, TKI, resistance, inhibitor

## Abstract

Lung cancer is treated with many conventional therapies, such as surgery, radiation, and chemotherapy. However, these therapies have multiple undesirable side effects. To bypass the side effects elicited by these conventional treatments, molecularly-targeted therapies are currently in use or under development. Current molecularly-targeted therapies effectively target specific biomarkers, which are commonly overexpressed in lung cancers and can cause increased tumorigenicity. Unfortunately, several molecularly-targeted therapies are associated with initial dramatic responses followed by acquired resistance due to spontaneous mutations or activation of signaling pathways. Acquired resistance to molecularly targeted therapies presents a major clinical challenge in the treatment of lung cancer. Therefore, to address this clinical challenge and to improve lung cancer patient prognosis, we need to understand the mechanism of acquired resistance to current therapies and develop additional novel therapies. This review concentrates on various lung cancer biomarkers, including EGFR, ALK, and BRAF, as well as their potential mechanisms of drug resistance.

## 1. Introduction

Lung cancer is known to cause more deaths than breast, prostate, and colon cancers combined, accounting for about 25% of all cancer deaths. The 5-year survival rate for all lung cancer patients is only about 18%, and roughly 85% of lung cancer cases are non-small cell lung cancer (NSCLC) [1]. In 2018, 234,030 lung cancer cases and 154,050 deaths are anticipated [2]. Due to both its high mortality and frequency in both sexes, developing novel and effective treatments for NSCLC is of major importance. Common treatments for early-stage NSCLC include surgical intervention, chemotherapy, and radiation therapy, all of which elicit undesirable side effects. Currently, molecularly-targeted therapies have become a research focal point due to their specificity to cancer cells and minimal adverse effects in comparison to conventional treatments [3]. A number of molecularly-targeted therapies focus on various receptor tyrosine kinases (RTKs) involved in cellular growth and survival.

RTKs are commonly mutated in NSCLC, leading to amplification of RTK signaling and activation of downstream and alternative signaling pathways which often converge on common downstream signaling effectors that elicit tumorigenesis [4]. Various RTKs, such as epidermal growth factor receptor (EGFR), hepatocyte growth factor receptor (HGFR/c-Met), and anaplastic lymphoma kinase (ALK), have been targeted for the development of therapeutics, as these are found to be often mutated in NSCLC patients [5]. v-Raf murine sarcoma viral oncogene homolog B1 (BRAF) is another potential target for advanced NSCLC treatment, since mutations in BRAF have been shown to play a role in tumorigenesis in NSCLC [6]. When mutated, these growth factor receptors could cause upregulation and amplification of various downstream signaling pathways, including the MAP kinase, PI3K/AKT, and mTOR pathways. These pathways are responsible for cell survival, proliferation, migration, and angiogenesis of cancerous cells [7].

Many of the current molecular therapies that target RTKs utilize tyrosine kinase inhibitors (TKIs), which act by binding to RTKs and inhibiting their respective signaling cascades, leading to inhibition of signaling pathways that would otherwise induce cell growth and proliferation (see Table 1) [5]. However, prolonged treatment with TKIs often results in the development of acquired drug resistance that limits the duration of their clinical benefit. A potential explanation for acquired TKI resistance may be due to acquisition of RTK mutations, epithelial-mesenchymal transition (EMT), and the activation of alternative signaling pathways [8]. Overcoming this resistance presents a challenge that has become an area of interest for many researchers. In this review, we summarize and discuss various biomarkers in NSCLC currently being targeted for therapy, their modes of resistance, and potential treatment options intended to delay or prevent resistance from occurring.

## 2. EGFR

**Figure 1:**
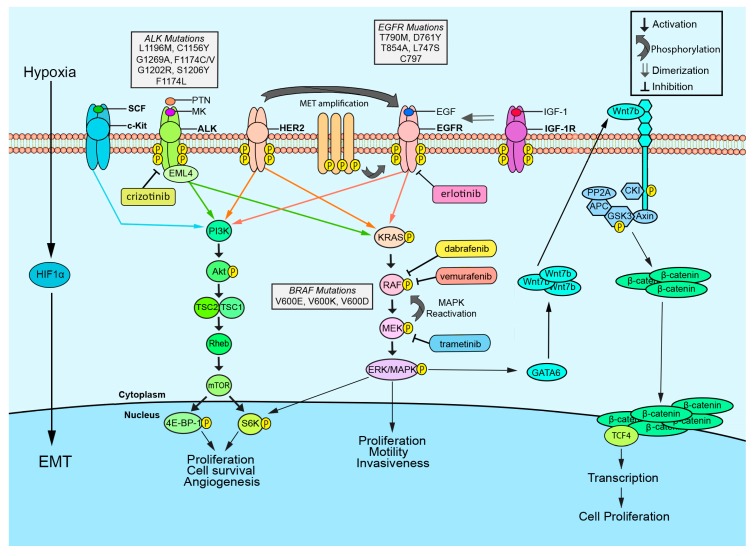
Mechanisms of resistance to molecularly targeted therapies in non-small cell lung cancer (NSCLC)**.** Phosphorylation of receptor tyrosine kinases such as ALK, c-MET, and EGFR lead to activation of downstream signaling pathways that are responsible for cell proliferation, survival, and angiogenesis. Receptor tyrosine kinase inhibitors (TKIs) inhibit receptor activation. However, due to mutations listed in the figure and activation of alternative signaling pathways, tumor cells acquire resistance against these TKIs. Activation of PI3K/mTOR, Wnt, and RAS-MAPK pathways may cause resistance to TKIs. Dimerization of IGF-1R with EGFR may activate the Akt and MAPK pathways despite inhibition.

Epidermal growth factor receptor (EGFR) is a transmembrane growth factor receptor which plays an important role in regulating cell proliferation, survival, and growth [54]. This receptor is usually present in its inactive form as a monomer. Upon binding to epidermal growth factor (EGF), EGFR dimerizes and autophosphorylates, leading to activation of downstream intracellular signaling cascades such as RAS-RAF-MEK-ERK and PI3K-AKT-mTOR (Figure 1) [24]. Mutations in EGFR that result in its constitutive activation are believed to be an important contributor to the tumorigenesis of many cancer types, including lung cancer. It has been reported that sensitizing mutations in the EGFR tyrosine kinase (TK) domain at exons 18–21 occur in about 15–18% of NSCLC patients, among which the L858R point mutation in exon 21 and a deletion in exon 19 (residues 747–750) constitute about 40% and 45% of EGFR mutations, respectively [55]. Exon 20 insertions account for roughly 9% of activating EGFR mutations, and prognosis for NSCLC patients with exon 20 insertion mutant EGFR is generally quite poor [56].

Tyrosine kinase inhibitors (TKIs) have been developed to inhibit mutated EGFR and its constitutive activation. The first generation of EGFR TKIs includes erlotinib and gefitinib. They reduce aberrant EGFR signaling via reversible inhibition of the ATP-binding pocket in the EGFR kinase domain. Unfortunately, the development of TKI resistance is common after prolonged use of these EGFR TKIs [9]. One major mechanism for the acquired resistance to EGFR TKIs is the development of the T790M secondary mutation within the EGFR kinase domain. The bulkier methionine residue at position 790 sterically hinders its interaction with TKIs and increases affinity for ATP, thus reducing inhibitor binding to the EGFR kinase domain while preserving catalytic activity [10]. Nevertheless, the T790M ‘gatekeeper’ mutation only attributes to 50% of all acquired resistance [57]. Therefore, a significant portion of acquired resistance arises from other mechanisms, such as upregulation of the mTOR pathway or EMT [58,59]. Additional secondary mutations such as D761Y, T854A, and L747S (Figure 1) are reported to arise subsequently to the EGFR TKI sensitizing L858R mutation and cause resistance [11].

In cell lines which are intrinsically resistant due to the presence of mutated EGFR, increased activity of TGF-β, which is often produced during an inflammatory response, has been shown to initiate transition to mesenchymal-like cell morphology accompanied by increased mobility, invasiveness, and resistance to erlotinib [60]. Inflammation also induces IL-6 secretion, which further bolsters resistance to erlotinib. It has been proposed that IL-6 upregulation corresponds with increased levels of Survivin, BCL-X1, and BCL-2 in the erlotinib-resistant cells, which suggests that IL-6 somehow protects cells from apoptosis [60,61]. Another study demonstrated *PIK3CA* mutations in 5% of lung cancer patients having *EGFR* mutations with acquired resistance. These mutations are reported to confer resistance by activating downstream targets such as AKT [62]. Our recent studies indicate that the activation of alternative signaling pathways, such as PI3K/mTOR and Wnt, may also cause resistance to EGFR TKIs in certain cell lines with wild-type EGFR; however, in cell lines with mutant EGFR, there is activation of the mTOR pathway. Activation of all these alternative pathways may contribute to EGFR and c-MET signaling, resulting in acquired resistance [63,64]. It has been shown that active β-catenin, which is a central downstream effector in the Wnt signaling cascade, is up-regulated in erlotinib-resistant cells, along with other proteins of the Wnt pathway. Furthermore, Wnt can cause activation of EGFR and MAPK signaling via the Wnt/Fz/LRP pathway [64,65].

**Figure 2:**
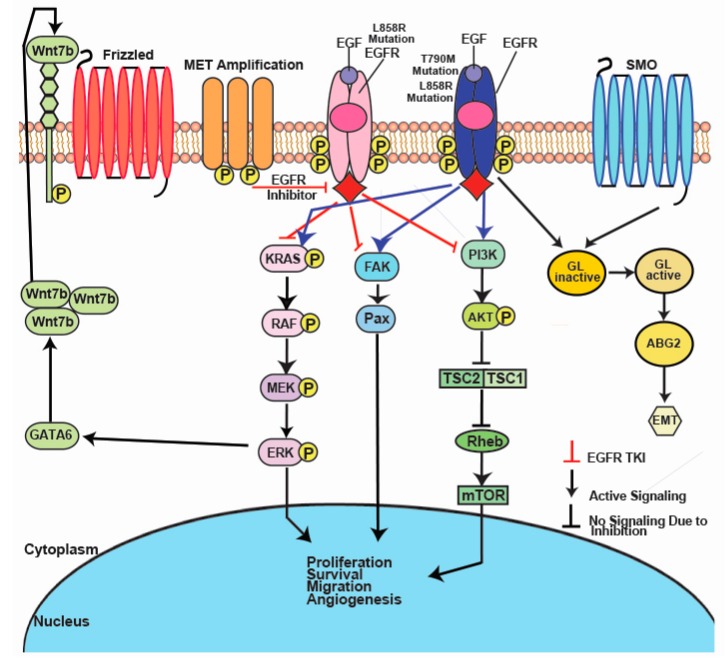
EMT, *MET* amplification, and IGF-1R confer resistance to EGFR TKIs. Dimerization with IGF-1R allows activation of the MAPK and PI3K pathways despite EGFR inhibition. *MET* amplification allows phosphorylation of EGFR and activation of downstream signaling. Activation of the Hedgehog signaling pathway and Wnt signaling pathways promote EMT, which may confer resistance to EGFR TKIs.

It has also been found that the hedgehog (Hh) signaling pathway is inappropriately activated in EGFR TKI resistant NSCLC cells, though silenced in EGFR TKI sensitive cells, implicating Hh activation in the development of EGFR TKI resistance via the induction of EMT and upregulation of the stem cell marker ABCG2 (Figure 2) [66]. The Hh pathway is a coordinator of many cellular processes, such as proliferation and differentiation, and it cooperates with the EGFR pathway during embryonic development of mammals to coordinate stem cell proliferation [67]. Abrogation of this pathway resulted in increased sensitivity of resistant NSCLC cells to EGFR TKI treatment, as well as decreased expression of ABCG2, further implicating its role in acquired resistance [66]. It has been shown that the G776^YVMA^ mutation in human epidermal growth factor receptor 2 (HER2) allows for the phosphorylation of EGFR receptors even in the presence of EGFR TKIs [68]. The knockdown of HER2 restored sensitivity to EGFR TKIs in H1781 lung cancer cells which have the G776^YVMA^ mutation, suggesting that inhibition of HER2 may prove a promising target to bypass EGFR TKI resistance [68]. Inappropriate amplification of *MET*, a gene that codes for an epithelial RTK that activates signaling of the MAPK, PI3K, and SRC pathways upon binding by its ligand, hepatocyte growth factor (HGF) [69], stimulates the AKT pathway and is also a major contributor to EGFR TKI secondary resistance (Figure 1). *MET* amplification causes overexpression of the MET receptor and activation of its downstream signaling pathways. In fact, *MET* gene amplification is shown to be one of the most relevant mechanisms responsible for the acquired resistance against EGFR TKIs (Figure 2) [70]. Crosstalk between c-MET and EGFR signaling pathways has also been observed [71,72]. Puri et al. have demonstrated that TKIs against c-MET and EGFR have a synergistic inhibitory effect on proliferation [72]. Furthermore, a recent study has also identified c-MET activation and upregulation of its associated ligand, HGF, as mediators of resistance to TKIs in VEGFR-mutant NSCLC. In this study, activation of the c-MET pathway also resulted in the formation of tortuous blood vessels within associated tumors [73]. More specifically, *MET* amplification has been shown to promote gefitinib resistance by activating PI3K through ERBB3 (HER3) despite EGFR inhibition via gefitinib [70]. Another study found inhibition of c-MET in *MET*-amplified NSCLC led to activation of the EGFR pathway [74]. Thus, EGFR/c-MET combination therapy could be a possible strategy to overcome c-MET resistance [75]. A recent study conducted by our laboratory found upregulation of mTOR and Wnt signaling proteins in c-MET/EGFR resistant NSCLC cell lines, implying the role of alternative cell signaling pathways in TKI resistance [63]. This suggests that a combination of Wnt and mTOR inhibitors with c-MET or EGFR inhibitors may improve the prognosis in NSCLC. However, further clinical trials are needed to confirm these results. Insulin-like growth factor 1 receptor (IGF-1R) is another RTK whose activation could contribute to acquired EGFR TKI resistance. IGF-1R has the potential to dimerize with EGFR after treatment with erlotinib, transducing signals to the AKT and MAPK signaling pathways that would otherwise be silenced by erlotinib (Figure 1). Inhibition of IGF-1R has been shown to enhance the effects of first generation EGFR TKIs, providing a possible route to overcome resistance [76].

Despite the development of resistance, first generation TKIs like erlotinib and gefitinib are still employed by physicians as a first line approach against lung cancer. Multiple recent studies suggest that the effectiveness of these drugs may be bolstered by combinatorial treatment with the diabetes drug Metformin. An earlier clinical observation study demonstrated that the use of Metformin in type 2 diabetes patients correlated with a lower risk of cancer, in general (hazard ratio 0.90), compared with use of sulfonylurea derivatives, which are also used for the treatment of diabetes [77]. At first, it was unknown whether Metformin truly had anticancer properties or if sulfonylurea derivatives simply increased the risk of cancer [77]. However, evidence strongly suggests that Metformin does indeed have anticancer properties and may be a viable cancer preventative and treatment supplement [61,78,79]. Metformin interacts synergistically with TKIs like erlotinib and gefitinib, and Metformin alone has been shown to reduce tumor size in xenografts and re-sensitize resistant cells to TKI treatments [78]. Metformin decreases IL-6 signaling, reverses EMT, and decreases activation of STAT3 and AKT [61,78]. Metformin also appears to activate JNK/p38 MAPK pathway and GADD153, which induces apoptosis and decreases proliferation in lung cancer cells [79]. Furthermore, Metformin has been shown to suppress TGF-β and bleomycin-induced pulmonary fibrosis exacerbation associated with gefitinib treatment via the inhibition of the TGF-β pathway [80]. Clinical trials are currently being done to study Metformin in combinatory treatments with an assortment of molecularly targeted therapies, including EGFR inhibitors, as well as its effect on overall survival and treatment-related toxicity in advanced stage non-small cell lung cancer patients.

The second-generation EGFR TKI, afatinib, is an irreversible inhibitor of EGFR. It was shown to have an EC_50_ against EGFR with the T790M mutation at a concentration of 9 nM [12], suggesting its potential to overcome TKI resistance due to the T790M mutation. In contrast to erlotinib and gefitinib, which solely bind to the ATP-binding site of EGFR, afatinib also forms covalent bonds with cysteine 797 and the cysteine residues of HER2 and ErbB-4, thus further inhibiting phosphorylation of EGFR [13]. In a Phase I study performed in patients with NSCLC, 12 patients were treated with afatinib at dosages of 20–50 mg per day. Six of the 12 patients had tumor size reductions; three achieved durable stable disease, including one with EGFR exon 19 and T790M mutations [12]. However, treatment with afatinib has been shown to phosphorylate STAT3 at tyrosine 705 and cause an increase in levels of RANTES mRNA. Activation of the STAT3 pathway has been implicated in development of acquired resistance in both erlotinib and gefitinib treatment, and thus resistance to afatinib can arise via this pathway. Treatment of afatinib in combination with a STAT3 inhibitor significantly inhibits growth, providing a route to overcome afatinib resistance in TKI-resistant cell lines [81]. Third generation TKIs have been designed to target both EGFR with activating mutations and T790M resistance mutation in NSCLC patients. Recently, the FDA has approved osimertinib (Tagrisso) or AZD9291, a third-generation EGFR TKI, as a breakthrough treatment for NSCLC patients whose tumors have a T790M mutation and whose disease has worsened after treatment with other EGFR inhibitors. Phase 1 clinical trials of osimertinib demonstrated a response rate of 61% and a disease control rate of 95% in advanced lung cancer patients with prevailing cancer progression after EGFR TKI treatment [14]. In April 2018, the FDA approved the use of osimertinib (AstraZeneca) as a first-line treatment for metastatic NSCLC patients with L858R mutations in exon 21 or deletions in exon 19 [15]. A recent Phase III FLAURA trial demonstrated the superiority of osimertinib over first-generation EGFR inhibitors gefitinib and erlotinib as a first-line treatment for EGFR-mutated NSCLC. Previously untreated EGFR-mutant NSCLC patients (556) from Asian populations were selected randomly to receive erlotinib, gefitnib, or osimertinib. Patients treated with osimertinib had a progression-free survival of 18.9 months compared to 10.2 months for patients treated with gefitinib or erlotinib. Median duration of response was also significantly greater in the osimertinib-treated patients compared to first-generation TKI treatment (17.2 vs. 8.5 months, respectively) [16]. Although mutant EGFR NSCLC patients bearing exon 20 insertions are generally unresponsive to first-line TKI therapy, recent studies suggest that osimertinib may prove effective as a therapy for patients with these activating mutations. Osimertinib was shown to inhibit growth of NSCLC cells bearing the most prevalent exon 20 insertion mutations, as well as inhibit tumor growth in tumor xenograft models bearing exon 20 insertions [17]. However, resistance to this TKI has already been reported, arising via mutations in the C797 EGFR codon and EGFR G796D, as well as amplified HER2 and MET signaling [18,19,20]. Treatment with selumetinib, a RAS-MAPK pathway inhibitor, in combination with osimertinib seemed to curb this resistance [82]. A recent study demonstrated that combinatorial treatment of a c-Met and ERBB inhibitor (capmatinib and afatinib) abolished tumor growth in NSCLC brain metastasis mouse models with *MET* amplification conferring acquired resistance to osimertinib [83]. Another study demonstrated high levels of *MET* amplification seen in tumor biopsies of a mutant T790M EGFR NSCLC patient following osimertinib treatment. Treatment with the MET inhibitor crizotinib conferred transient symptomatic improvement of this patient, suggesting MET inhibition may be a potential treatment option for patients progressing on osimertinib treatment with *MET* amplification [84]. Another third generation TKI, rociletinib, was designed to inhibit the activating EGFR mutation and the T790M resistance mutation, with a focus on sparing wild-type EGFR. This design was shown to be effective in preclinical models in which rociletinib was found to have significantly less activity against wild-type EGFR than the early-generation TKIs currently being used clinically, as well as AZD9291 [21]. However, due to the adverse effects and limited response rates following treatment with rociletinib, the FDA has currently not approved this drug for treatment of NSCLC patients [22].

A fourth generation TKI, EAI045, has been developed as a novel EGFR inhibitor to overcome both C797 and T790M mutations that confer resistance to third generation inhibitors. EAI045, in contrast to other EGFR TKIs, acts via allosteric inhibition rather than competitive inhibition of the ATP-binding site. The growth inhibitory activity of EAI045 increases when combined with cetuximab, an EGFR inhibitor that prevents EGFR from dimerizing. A combination of EAI045 and cetuximab demonstrated significant tumor growth inhibition in mouse models carrying the T790M mutation, whereas treatment with EAI045 alone failed to exhibit any decrease in tumor growth. Additionally, since dimerization-defective/independent mutants were markedly more sensitive to EAI045, it is hypothesized that EAI045 acts upon a single component of the EGFR dimer [20,23]. Thus, EAI045 treatment appears to be a promising therapeutic strategy in NSCLC patients with resistance to first, second, and third generation EGFR TKIs. Patients being treated with EGFR inhibitors will require continual assessment of progression of lung cancer in order to study how these patients become resistant to therapy and to develop strategies to prevent resistance.

## 3. ALK

The anaplastic lymphoma kinase (ALK) is a receptor tyrosine kinase encoded by the *ALK* gene localized on chromosome 2 and is often associated with lung cancer when mutated. This kinase is typically expressed in the central and peripheral nervous systems [85,86]. ALK is reported to regulate several different pathways involved in cellular proliferation and survival, such as PI3K-AKT-mTOR, RAS-RAF-MEK-ERK, and the JAK-STAT pathway, once it dimerizes and is activated by autophosphorylation after binding with its ligands, pleiotrophin (PTN), and midkine (MK) [87,88] (Figure 1). In lung cancer patients, *ALK* gene amplification, mutation, and rearrangement is known to be associated with tumor development [88,89,90], and around 5% of NSCLC cases are diagnosed with an *ALK* gene rearrangement [90].

Crizotinib, a first generation ALK TKI, has been approved by the FDA for treating locally advanced or metastatic ALK-positive NSCLC tumors by competitively binding to the ATP-binding site [25]. Due to acquired resistance to crizotinib, the efficacy of this TKI is limited to approximately 1 year. Secondary mutations in the *ALK* gene are considered to be the most frequent mechanisms mediating resistance to ALK inhibitors, such as crizotinib. Point mutations such as L1196M, C1156Y, F1174L, and F1174V (Figure 1) in the ALK kinase domain have been found in NSCLC patients treated with crizotinib. These mutations render crizotinib less effective by decreasing ligand affinity for its active site [24,26,27]. A recent study reported that the F1174V mutation may induce secondary crizotinib resistance via an alteration in secondary structure of the protein, resulting in a conformational change within the ALK kinase domain and thus reducing crizotinib affinity and efficacy [28]. The gatekeeper mutation L1196M in ALK kinase domain confers resistance to crizotinib [24,29] by concurrently increasing and decreasing the binding affinities of ATP and ALK inhibitors, respectively. The other secondary mutation C1156Y causes resistance by inducing a marked allosteric effect on the ATP-binding site of the ALK kinase domain [30]. Another study reported the presence of L1196M and G1269A point mutations of the crizotinib target site in four out of eleven patients treated with crizotinib [27]. Katayama et al. identified new mutations such as G1202R, S1206Y, and 1151Tins (Figure 1) in four of eighteen ALK-positive NSCLC patients who had progressed on crizotinib [26]. Additionally, in parallel with mutations, copy number gain of the *ALK* gene rearrangement has been shown as a potential mechanism of resistance to crizotinib. The copy number gain was observed due to more copies of the ALK rearrangement per cell and an increase in cells displaying the rearrangement pattern in the patients treated with crizotinib [26,27].

Recently, Wilson et al. conducted a large-scale functional genomics study using a genome-scale lentiviral expression library of human open reading frame (ORF) to identify novel genes conferring resistance to ALK inhibitors, crizotinib, and TAE684 (a second-generation ALK inhibitor). This study demonstrated that the neuregulin-1 and P2Y purinergic G-protein coupled receptor proteins mediate resistance to the ALK inhibitors, TAE684 and crizotinib, in lung cancer [31]. Some other studies focusing on the mechanism of resistance to crizotinib have shown involvement of mutations in *EGFR* and *KRAS* [27], activation of the ErbB family through phosphorylation [26], EMT [91], activation of the insulin-like growth factor 1 receptor (IGF-1R) pathway [92], *KIT* amplification [26], and autophagy [93]. A recent study also performed a multi-phospho-RTK antibody array to screen for secondary RTK involvement in ALK inhibitor resistance within two crizotinib-resistant and one TAE684-resistant cell line, further implicating EGFR, IGF-1R, and HER, as well as MET, in acquired ALK inhibitor resistance [94].

Ceritinib, alectinib, and brigatinib (AP26113) are amongst the second generation of ALK TKIs which have been developed to overcome resistance to first generation TKIs conferred by ALK kinase domain mutations [33,34]. Brigatinib has shown efficacy in first generation TKI-resistant ALK mutants carrying C1156Y, I1171S/T, V1180L, L1196M, L1152R/P, E1210K, and G1269A mutations, and it has demonstrated therapeutic potency following crizotinib treatment [43]. Metastasis into the central nervous system is a common complication of NSCLC patients carrying mutant ALK undergoing treatment with crizotinib, and second generation TKIs, namely alectinib, have demonstrated therapeutic activity in treating central nervous system metastasis in ALK-positive NSCLC patients [95]. Furthermore, alectinib was recently shown to be more effective and less toxic than crizotinib in treating ALK-positive NSCLC as a first line therapy, demonstrating a 12-month event-free survival rate of 68.4% in comparison to 48.7% with crizotinib [38]. However, there are other mutations which have arisen that limit these next-generation inhibitors, as well. As with the first generation of TKIs, specific *ALK* gene mutations confer resistance to the various second generation TKIs, such as G1202R, I1171T/N/S, and V1180L for alectinib, and G1202R and F1174C/V (Figure 1) for ceritinib [35,39,40,41,42]. Additionally, several studies have shown involvement of hepatocyte growth factor (HGF)/MET signaling pathway activation in the acquired resistance to alectinib [96,97].

A few other studies have reported that EGFR ligands [98] and hypoxia-induced EMT confer resistance to alectinib [91]. It has been shown that HGF, a ligand of MET, induces resistance to alectinib, but not to crizotinib, via activation of MET signaling in NSCLC cell lines [97,98]. Recently, Toyokawa et al. reported a novel ALK G1123S mutation in ALK-positive NSCLC patients acquiring resistance to ceritinib [36]. Another in vitro study on a human-derived neuroblastoma cell line (SH-SY5Y), with G1123S or G1123D mutations at codon 1123, showed resistance to an ALK inhibitor, TAE684 [32]. A novel T1151K mutation has also been described in a patient that demonstrated cancer progression following treatment with crizotinib and ceritinib [37]. These mutations could either sterically block ATP binding and/or alter the glycine-rich loop dynamics, perturbing interactions with ALK inhibitors that require a specific conformation of the loop for binding [32]. Additionally, one recent study showed reactivation of the RAS-MAPK pathway (Figure 1) as a novel mechanism of acquired resistance in ALK-positive lung cancers [99]. This study also identifies new potential biomarkers of ALK-inhibitor resistance and provides a potential explanation for the role of the upstream EML4 (echinoderm microtubule-associated protein like 4) fusion partner in driving chronic ALK signaling. This study also demonstrates that a combination of both ALK and the RAS-MAPK component MEK inhibitors could be a potential approach to overcome resistance and improve the prognosis of ALK-positive lung cancer [99].

Third-generation ALK inhibitors have been under development to overcome second-generation inhibitor resistance in ALK-positive NSCLC patients. Amongst these molecules are lorlatinib (PF-06463922), entrectinib (RxDx-101), and ensartinib (X-398). Lorlatinib serves as an ATP-competitive TKI for both ALK and ROS1, an RTK in the insulin receptor family with striking similarity to ALK that is overexpressed in several cancer types, but absent in normal lung tissue. *ROS1* rearrangement is present in 1–2% of NSCLC cases, and detection of these rearrangements is critical in selecting appropriate inhibitor-based treatment for NSCLC [100]. Lorlatinib has also demonstrated efficacy in treating ALK-positive NSCLC metastases to the central nervous system. As part of Phase 1–2 clinical trials, 54 ALK or ROS1-mutation positive patients were treated with lorlatinib, demonstrating an overall response rate of 50%. Common adverse effects were limited to hypercholesterolemia and peripheral edema [44]. Entrectinib serves a three-fold role as an inhibitor of ALK and ROS1, as well as NTRK1–3 fusion proteins, which are receptor tyrosine kinases that control synaptic strength and plasticity in mammals. It has demonstrated therapeutic potential in a wide array of cancer types and is currently undergoing Phase 1–2 clinical trials [46]. Ensartinib is another novel ALK inhibitor undergoing clinical trials. In Phase 1–2 clinical trials, 80 NSCLC patients carrying the ALK mutation were given 225 mg of ensartinib daily for 28 days. Partial response rate for evaluable patients was 58%, and 20% of patients reached stable disease. In patients who had received crizotinib treatment previously, a partial response of 64% was obtained, with 27% of patients reaching stable disease [47]. However, as with the other generations of TKIs, novel mutations quickly arise that confer resistance to these third generation inhibitors after prolonged treatment.

Resistance to lorlatinib has recently been identified to occur through a L1198F mutation. This mutation confers resistance via steric interference at the active site, preventing drug binding. Interestingly, however, this mutation counteracts the effect of the C1156Y mutation that elicits resistance to crizotinib, thus resensitizing the cancer to crizotinib treatment [45]. Studies regarding the mechanisms of resistance against entrectinib in NSCLC are limited. However, mutations that result in drug resistance seem to be localized to the NTRK1–3 catalytic domains [101]. Another study identified a mutation in NTRK3 that likewise resulted in drug resistance in mammary analogue secretory carcinoma [102]. Such mutations may have a similar effect in NSCLC, as well. Mechanisms of resistance regarding ensartinib remain under thorough investigation. Continued research is required to elucidate the mechanism of acquired resistance for current small molecule inhibitors targeting ALK, as well as to develop effective therapies to bypass this resistance, possibly by targeting secondary RTK involvement.

## 4. BRAF

BRAF (v-Raf murine sarcoma viral oncogene homolog B1) is a member of the RAF serine/threonine protein kinases family. BRAF encodes for a RAF kinase that signals downstream of RAS to activate the MAPK pathway, which causes uncontrolled growth and differentiation upon mutation [103]. BRAF is the most commonly mutated gene in melanoma; however, BRAF mutations have also been shown to be associated with NSCLC with a frequency of approximately 2–3% of cases. In melanoma, valine 600Glu (V600E) mutation within exon 15 of the kinase domain constitutes more than 90% of mutations in melanoma. In lung cancer, the V600E mutation is the most common mutation, yet they also occur at other positions within the kinase domain. A study reported BRAF mutations in 18 (3%) out of 697 patients with lung adenocarcinoma. V600E BRAF mutation was observed in 50% of these 18 patients, while G469A and D594G were found in 39% and 11%, respectively [104]. Another recent study reported seven out of 273 NSCLC cases (2.6%) with BRAF mutations, of which 58% (four out of seven) were having V600E, while others were reported with K601N, L597Q, and G469V mutations [105]. A V600 mutation on exon 15 totals 50% of all BRAF mutants, while non-V600 BRAF mutations compose the remaining 50% [104].

Recently, promising results from a Phase 2 clinical study with dabrafenib, a BRAF inhibitor, has shown efficacy in the treatment of advanced NSCLC patients with V600E BRAF mutation (Figure 1) [48]. However, another study has found acquired resistance to dabrafenib in a patient after 8 months of treatment. The primary mechanism responsible for this acquired resistance to dabrafenib in this patient has been reported to be a G12D mutation in KRAS (Figure 1) [49]. Subsequent studies need to be carried out to further understand the therapeutic potential of BRAF inhibitors for the treatment of NSCLC patients.

**Figure 3:**
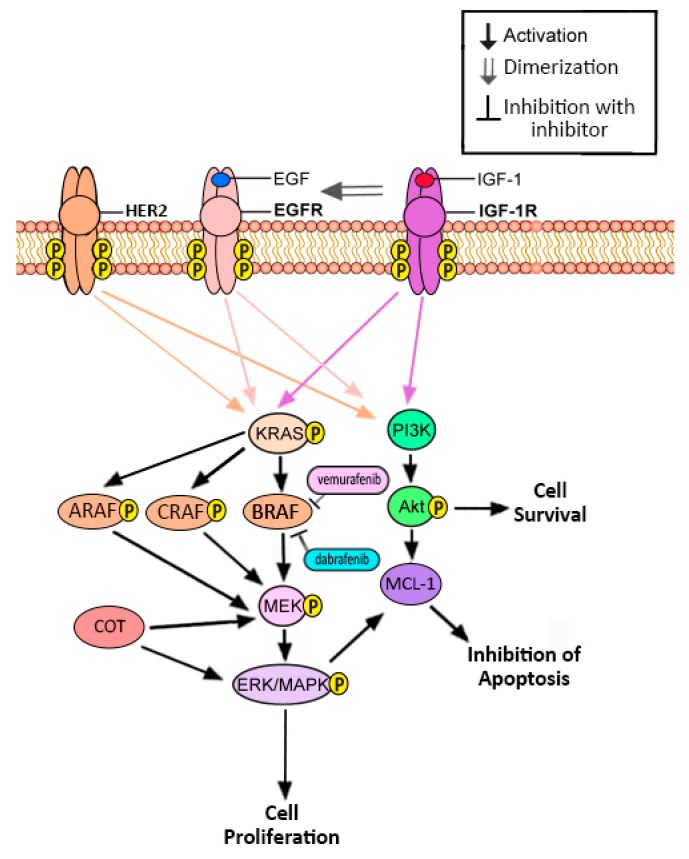
CRAF, ARAF, COT, and MCL-1 confer resistance to BRAF inhibitors. Elevated expression of alternative RAF isoforms (ARAF and CRAF), as well as MAP3K8/COT, can stimulate the MAPK pathway despite BRAF inhibition. The PI3K and MAPK pathways also activate MCL-1 and may provide a route to escape apoptosis and bypass BRAF inhibition.

Mutant BRAF in NSCLC is known to display resistance towards current inhibitors after prolonged treatment. A BRAF oral inhibitor, vemurafenib, is effective in the treatment of advanced stages of melanoma with the V600E mutation (Figure 1). However, in NSCLC, vemurafenib provides little to no response. The research conducted on inhibitors of mutant BRAF in NSCLC is limited; however, extensive work has been done on elucidating mechanisms of resistance to BRAF inhibitors in melanoma and may provide parallels to mechanisms in lung cancer. One study has shown that elevated expression of alternate isoforms of RAF proteins (CRAF and ARAF) can continually stimulate the MAPK pathway despite BRAF inhibition, providing a mechanism of resistance (Figure 3) [50]. Increased expression of MAP3K8 or COT can stimulate the MAPK pathway to bypass BRAF inhibition (Figure 3) [50,51]. The MAPK pathway and PI3K pathways also activate MCL-1, an antiapoptotic factor. Parallel activation of the PI3K pathway may inhibit apoptosis in BRAF-mutant NSCLC through MCL-1 despite inhibition of BRAF [50]. Trametinib, a MEK inhibitor, is used in conjunction with vemurafenib to enhance the efficacy (Figure 1). There have been promising results with the combination of vemurafenib and trametinib with an increase in upregulation BIM, a pro-apoptotic protein, in V600E and non-V600E cells which plays a major role in apoptosis [52]. In a recent study, the combination of trametinib and dabrafenib resulted in increased growth inhibition by increased caspase3/7 activity in H166 and H508 cell lines. This combinatory treatment also increased ERK inhibition compared to monotherapy [53]. These results suggest that combinatorial therapy may be an effective route to overcome resistance to treatment in BRAF-mutant NSCLC.

## 5. Conclusions

Resistance against current molecularly-targeted therapies represents a major clinical challenge in NSCLC. A plethora of research is ongoing to understand the mechanism of this acquired resistance by tumor cells. Mechanisms of resistance for several TKIs against EGFR, c-MET, ALK have been investigated and identified. These mechanisms include the attainment of mutations in the drug target and the activation of alternative signaling pathways, and further drug development is underway in order to overcome these modes of acquired resistance. Further studies are also necessary to further elucidate the mechanism of acquired resistance to HER2 and BRAF, as well as to develop effective therapies to overcome them. Gaining insight into the mechanisms underlying tumor cell drug resistance is critical for developing more effective therapies [106].

Combinatorial strategies could be effective in overcoming TKI resistance in lung cancer and have already shown some promise, such as in BRAF-mutant NSCLC. To achieve optimal efficacy, these strategies are directed at both mutations in native target sites as well as alternative pathways [63]. However, increased drug toxicity may be a factor that limits the use of combination therapies. In addition, tumors in different patients may acquire resistance via different mechanisms. Thus, it is important to evaluate each patient’s unique mechanism of resistance at the molecular level in order to tailor targeted therapies to individual patients. One approach involves repeated tissue biopsies to determine genomic evolution as a result of therapy. However, this method requires high level of patient compliance, is rather invasive, and can be confounded by intra-tumor heterogeneity [107]. To combat these issues, technologies are being developed to allow for sampling of patients’ blood for analysis of circulating tumor cells or plasma for circulating cell-free tumor DNA, which allow a more homogenous sample of tumor genetic material, due to the presence of a significant amount of circulating tumor DNA in the blood, that is far less invasive and easily-repeatable [108]. Novel gene mutations, single nucleotide polymorphisms, and copy number variations, which are found to contribute to the development of drug resistance in tumor cells, could also prove valuable as a target to develop newer personalized therapies which, in effect, would reduce patient morbidity [109,110,111]. Genomic and functional genomic approaches such as systematic analyses of genotype-specific drug responses and large-scale cell line genomic profiling are currently being employed on tumors or cancer cell lines to find these mechanisms of drug resistance [111,112].

Altogether, many avenues combating treatment resistance in lung cancer patients are currently being developed and explored, leading to a promising future for the advancement of molecular targeted therapy. Lung cancer is a disease with high molecular complexity. Hence, newer genomic approaches like next-generation sequencing are needed to understand the entire drug-resistance repertoire for better treatment of lung cancer subjects in a more personalized way.

## Figures and Tables

**Table 1:** Current molecularly-targeted therapeutics, their associated targets, and acquired mutations conferring resistance.

Inhibitor	Target	Acquired Mutations Conferring Resistance	References
Erlotinib	EGFR	T790M, D761Y, T854A, L747S	[9,10,11]
Gefitinib	EGFR	T790M, D761Y, T854A, L747S	[9,10,11]
Afatinib	EGFR, HER2	T790M	[12,13]
Osimertinib (AZD9291)	EGFR	C797, G796D	[14,15,16,17,18,19,20]
Rociletinib	EGFR	C797	[21,22]
EAI045	EGFR	Under Investigation	[20,23]
Crizotinib	ALK, MET, ROS1	L1196M, C1156Y, F1174L, F1174V T1151K	[24,25,26,27,28,29,30]
TAE684	ALK	G1123S, G1123SD	[31,32]
Ceritinib	ALK	G1202R, F1174C/V, G1123S, T1151K	[33,34,35,36,37]
Alectinib	ALK	G1202R, I1171T/N/S, V1180L	[34,38,39,40,41,42]
Brigatinib (AP26113)	ALK, ROS1	Under Investigation	[34,43]
Lorlatinib (PF-06463922)	ALK, ROS1	L1198F	[44,45]
Entrectinib (RxDx-101)	ALK, ROS1, NTRK1–3	NTRK1, NTRK2, NTRK3	[46]
Ensartinib (X-398)	ALK, ROS1, MET, SLK	Under Investigation	[47]
Dabrafenib	BRAF	G12D KRAS	[48,49]
Vemurafenib	BRAF	Alternate isoforms of RAF proteins	[50,51,52]
Trametinib	MEK	Under Investigation	[52,53]
